# Learning from national implementation of the Veterans Affairs Clinical Resource Hub (CRH) program for improving access to care: protocol for a six year evaluation

**DOI:** 10.1186/s12913-023-09799-5

**Published:** 2023-07-25

**Authors:** Lisa V. Rubenstein, Idamay Curtis, Chelle L. Wheat, David E. Grembowski, Susan E. Stockdale, Peter J. Kaboli, Jean Yoon, Bradford L. Felker, Ashok S. Reddy, Karin M. Nelson

**Affiliations:** 1 Evidence-Based Practice Center, RAND Corporation, Santa Monica, CA USA; 2 Geffen School of Medicine and Fielding School of Public Health at UCLA, Los Angeles, CA USA; 3 Primary Care Analytics Team, VA Puget Sound Healthcare System, Seattle, WA USA; 4 The Department of Health Systems and Population Health in the School of Public Health, University of Washington, Seattle, USA; 5 VA HSR&D Center for the Study of Healthcare Innovation, Implementation, and Policy, VA Greater Los Angeles Healthcare System, Los Angeles, USA; 6 Department of Psychiatry and Biobehavioral Sciences, University of California, Los Angeles, USA; 7 Center for Access and Delivery Research and Evaluation (CADRE), Iowa City VA Healthcare System, Iowa City, IA USA; 8 Health Economics Resource Center, VA Palo Alto Health Care System, Menlo Park, CA USA; 9 Department of General Internal Medicine, UCSF School of Medicine, San Francisco, CA USA; 10 Mental Health Service Line, VA Puget Sound Healthcare System, Seattle, WA USA; 11 Department of Psychiatry and Behavioral Sciences, University of Washington School of Medicine, Seattle, WA USA; 12 Department of Medicine, University of Washington School of Medicine, Seattle, WA USA

**Keywords:** Access to Care, Staffing, Learning Organizations, Telehealth, Primary Care, Mental Health Care, Implementation Science

## Abstract

**Background:**

The Veterans Affairs (VA) Clinical Resource Hub (CRH) program aims to improve patient access to care by implementing time-limited, regionally based primary or mental health staffing support to cover local staffing vacancies. VA’s Office of Primary Care (OPC) designed CRH to support more than 1000 geographically disparate VA outpatient sites, many of which are in rural areas, by providing virtual contingency clinical staffing for sites experiencing primary care and mental health staffing deficits. The subsequently funded CRH evaluation, carried out by the VA Primary Care Analytics Team (PCAT), partnered with CRH program leaders and evaluation stakeholders to develop a protocol for a six-year CRH evaluation.

The objectives for developing the CRH evaluation protocol were to prospectively: 1) identify the outcomes CRH aimed to achieve, and the key program elements designed to achieve them; 2) specify evaluation designs and data collection approaches for assessing CRH progress and success; and 3) guide the activities of five geographically dispersed evaluation teams.

**Methods:**

The protocol documents a multi-method CRH program evaluation design with qualitative and quantitative elements. The evaluation’s overall goal is to assess CRH’s return on investment to the VA and Veterans at six years through synthesis of findings on program effectiveness. The evaluation includes both observational and quasi-experimental elements reflecting impacts at the national, regional, outpatient site, and patient levels. The protocol is based on program evaluation theory, implementation science frameworks, literature on contingency staffing, and iterative review and revision by both research and clinical operations partners.

**Discussion:**

Health systems increasingly seek to use data to guide management and decision-making for newly implemented clinical programs and policies. Approaches for planning evaluations to accomplish this goal, however, are not well-established. By publishing the protocol, we aim to increase the validity and usefulness of subsequent evaluation findings. We also aim to provide an example of a program evaluation protocol developed within a learning health systems partnership.

**Supplementary Information:**

The online version contains supplementary material available at 10.1186/s12913-023-09799-5.

## Background

Published protocols for large-scale program evaluations have the potential to improve the quality and usefulness of subsequent evaluation results [1]. This paper describes the evaluation protocol for assessing national implementation of the Department of Veterans Affairs’ (VA’s) innovative Clinical Resource Hub (CRH) program.

In brief, CRH aims to improve patient access to local outpatient care by implementing regionally organized contingency primary care and mental health staffing support to outpatient care sites, primarily through telehealth modalities. By contingency staffing, we mean time-limited staffing to cover staffing vacancies. To accomplish this, CRH aims to implement 18 regional VA hubs that employ contingency staff to remediate vacancies across VA’s more than 1000 outpatient sites, using primarily virtual care modalities [2]. While temporary employment agencies have long been used by industries to meet short-term staffing needs, and more recently have been used to address temporary nursing [3] or physician [4, 5] staffing gaps, we found no prior scientific publications describing healthcare organization implementation or evaluation of large-scale, internally staffed, regionally organized contingency staffing support for geographically dispersed outpatient sites. The CRH evaluation focuses on assessing whether and how CRH achieves its implementation and outcome goals.

The VA healthcare system is one of the largest managed care systems in the United States, providing care nationally to over 9 million military Veterans across all 50 United States and US territories [2] under the direction of the United States Congress [6]. The VA system provides comprehensive health services including primary care, mental healthcare, and specialty care through a regional administration system of 18 Veterans Integrated Service Networks (VISNs) that operate 171 medical centers and more than 1000 Community Based Outpatient Clinics (CBOCs). Medical centers manage far-flung networks of CBOCs, rehabilitation units, nursing homes, and other facilities [2]. VA is a learning organization and as such funds a large health services research program that includes investigator-initiated research, quality improvement research [6] and VA operations-partnered research [7]. Among othe partnership research priorities, VA aims to rigorously evaluate all new national programs including CRH.

The CRH program was initially implemented through VA-wide policy change. In 2018 the United States Congress mandated that VA focus on improving access to care through better support for staffing deficits [8], as well as through other program improvements such as increased access to non-VA community resources [2]. In response, among other improvement initiatives, VA leaders initiated CRH planning. In October 2019, the VA Office of Primary Care (OPC) began implementing CRH, based on a directive to all 18 VA administrative regions (Veterans Integrated Service Networks or VISNs) and their over 1000 VA outpatient sites. The directive built on three prior pilot projects that tested providing regional telehealth staffing support, [9–12] as well as on existing VA telehealth capabilities [9]. In August 2020, OPC requested that its Primary Care Analytics Team (PCAT), an embedded research and evaluation unit, [13] plan a comprehensive six year evaluation of the national CRH program.

Publication of protocols for program evaluations is less common and less standardized than publication of protocols for randomized trials but may be increasingly important as healthcare delivery systems and their funders take steps to become learning health systems [14, 15]. Our protocol development process aimed to foster scientific consensus across multidisciplinary researchers as well as tightly connecting researcher and clinical operations partner goals. We expect that our protocol will continue development over the duration of the project as a living document that serves these needs.

The CRH evaluation is built on the initial CRH program goals, rationale, implementation strategies, and structure, and calls for documenting these over time. The protocol also calls for multi-method evaluation of the types, quality, and effectiveness of CRH services, as well as their cost and acceptability to stakeholders, and includes an initial formative evaluation focus followed by a summative evaluation of program effectiveness and value.

The evaluation protocol relies on the RE-AIM (Reach, Effectiveness, Adoption, Implementation, and Maintenance) [16, 17] framework as the foundation for a logic model, specific aims, evaluation questions, and data collection and analysis plans.

The objectives for developing the CRH evaluation protocol were to prospectively: 1) identify the outcomes CRH aimed to achieve, and the key program elements designed to achieve them; 2) specify evaluation designs and data collection approaches for assessing CRH progress and success, including return on investment relative to program goals; and 3) guide the activities of five geographically dispersed evaluation teams.

## Methods

### Overview of the CRH Program

The CRH program aims to implement centralized units, termed “hubs,” within each of the 18 VA administrative regions (VISNs). Hubs are expected to provide timely short-term staffing support for up to two years to VA outpatient sites within their regions that experience staffing vacancies in front-line primary or mental health care clinical teams. Hub clinicians rely mainly on telehealth care (telephone; home or in-clinic video; or secure messaging) to provide direct clinical primary and mental health care to enrolled Veterans in geographically dispersed VA outpatient sites within their regions. All outpatient sites in the region may request hub assistance; those sites that receive hub help are termed “spoke” sites. Program developers anticipated that many spoke sites would be in medically underserved rural areas.

### Overview of the CRH Evaluation

The evaluation is designated as quality improvement by and under the purview of OPC and its Executive Director and supports the CRH program’s requirements to report findings to its Advisory Board and to Congress. The evaluation is funded on a yearly basis by OPC, with an anticipated duration of six years (October 2020 through September 2026), when CRH stakeholders will make summative decisions about whether or how to maintain or expand the program.

### CRH Program rationale

The VA system problem that CRH was developed to address was observed geographic variations in access to care as expressed in the US Congressional Maintaining Internal Systems and Strengthening Integrated Outside Networks (MISSION) Act for VA enacted in 2018 [8, 10]. The rationale for developing the CRH program in response to the MISSION Act was that remediating short-term staffing deficits in primary care and mental health had the potential to reduce patient access variations [18, 19].

The CRH program rationale builds on the concept of contingency staffing [20–23]. Contingency staffing is the organized availability of temporary or short-term personnel to replace absent employees or to meet high demand and is a cornerstone for ensuring continuously optimal access [9]. Widely used in industry and healthcare, contingency staffing can avoid the necessity to chronically overstaff in order to cover temporary but critical staffing deficits [24–27]. Standard staffing guidelines for primary and mental health care in VA and other systems are calculated to include sufficient staff for coverage of routine staff absences such as vacations or sickness and for routine increases in demand (e.g., yearly vaccination clinics). Non-routine, time-limited gaps in staffing, however, are the focus of CRH. These gaps are due to problems such as unexpected staff turnover, prolonged absences, or need for work adaptations and may not be feasible to cover with existing staff. Human resources-based hiring delays or locally insufficient availability of healthcare professionals often compound these issues, resulting in sustained impacts on patient access to care. Literature on contingency staffing [28, 29] suggests that availability of primary care and mental health contingency staff could reduce negative impacts of staffing losses on both patients and on clinical care providers and teams.

### CRH Implementation strategy

At baseline and during the initial implementation year, the main strategies used to implement CRH included mandating program elements (see CRH Roadmap, Appendix 1) and engaging regional administrative leadership. Strategies for engagement included initial and periodic strategic planning with stakeholders, development of written instructional information, nationally based training sessions for CRH hub leaders and staff, and ongoing collection of feedback from regional leaders and from formative evaluation data. Methods also included development of online tools for regional hubs to use to report on progress and for spoke sites to use for requesting staffing support. Finally, at the time of program initiation, regional administration could either use staff or funding shifted from other uses or could apply for additional funds from VA operations leadership. As shown below, the evaluation protocol includes data collection plans for documenting these key elements of the implementation process as they evolve over time [30].

### Evaluation structure

**Fig. 1:**
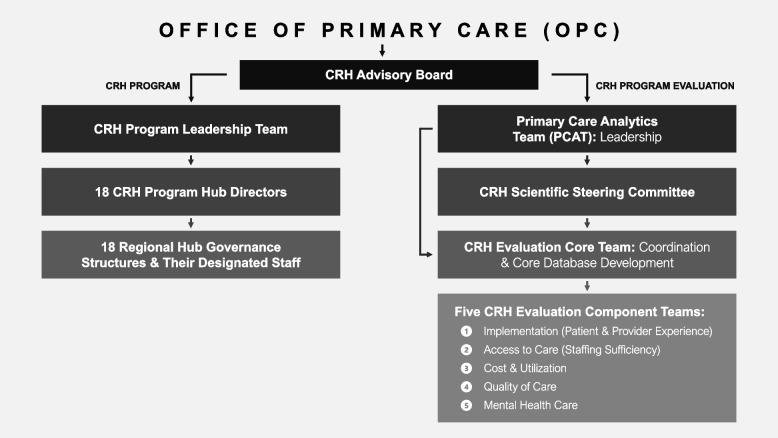
Organizational structure of the Clinical Resource Hub (CRH) program and CRH evaluation within the veterans administration’s office of primary care

Figure 1 shows the five CRH evaluation teams and their key roles, as well as the evaluation’s reporting structure. OPC leads implementation of CRH. Both the CRH program and the evaluation are overseen in addition by a multi-stakeholder CRH Advisory Board convened by OPC. The evaluation is carried out by PCAT [13] and its affiliated investigators and teams under PCAT leaders (authors KN, CW and IC). To develop the protocol, PCAT solicited proposals from formally affiliated PCAT health services research teams located in geographically disparate VA Health Services Research and Development Centers. Authors BF, PK, AR, SS, JY each lead one of the five CRH evaluation component teams shown in Fig. 1. The CRH Evaluation Core Team (including authors IC, CW, AR, LR, and DG and others) coordinates across the five component teams, including coordinating development and maintenance of a unified protocol. The Core Team also assembles and documents a central CRH data base across all teams. PCAT additionally convened a CRH Scientific Advisory Group (including author DG) to provide input on scientific aspects of the evaluation.

### Evaluation protocol development process

Protocol development began in late 2019 in anticipation of funding; the resulting protocol covering the first evaluation year was approved by OPC in April 2020. The protocol covering years two through six was approved in April 2021. The evaluation core team supported regular interaction between intervention and evaluation stakeholder groups, including all evaluation teams, through weekly or monthly teleconferences and yearly CRH Scientific Steering Committee meetings. The five evaluation teams used these interactions to develop successive written evaluation plans that the core team integrated into the evaluation protocol. The core team used two online surveys (2019, 2020) of CRH program leaders and evaluation team leaders to prioritize evaluation questions and to solidify scientific roles among the teams.

### Evaluation protocol overview

The initial 2020 approved evaluation protocol included a logic model intended to link key program elements with needed evaluation data It also included specific aims built around the RE-AIM framework, [16, 17] with accompanying evaluation questions. The 2020 protocol included revised versions of these and added data collection and analysis plans and timelines.

### CRH Program evaluation logic model

**Fig. 2:**
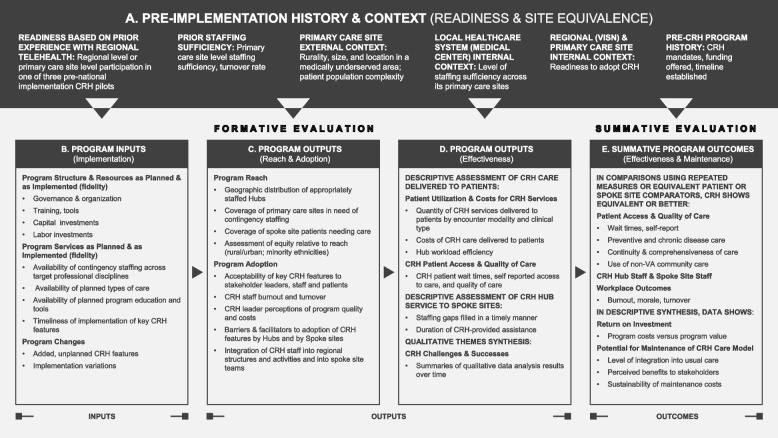
The Clinical Resource Hub (CRH) Evaluation logic model

The logic model provided a conceptual framework for evaluating the CRH program that shows a progression from intervention design to outputs and outcomes. The model (Fig. 2) was built first on CRH elements identified in the program’s original roadmap (Appendix 1) and implementation plans. Additional information came from CRH documents; from interviews with CRH leaders regarding goals and expected outcomes; [31] and from literature on contingency staffing. The logic model’s format is adapted from the R&D logic model tested by Park [32] and identifies needed evaluation data within the categories inputs, outputs, outcomes and context. The model integrates domains from the RE-AIM evaluation framework [16, 17]) into the four categories.

### Logic model domains

Figure 2 columns A – E show the logic model as (A) pre-implementation history and context; (B) program inputs (implementation); (C) program outputs (reach and adoption); (D) program outputs (effectiveness); and (E) summative outcomes (effectiveness). Columns A, B, C, and D are part of formative evaluation, showing observational data that will be collected for the first four years of the evaluation and serially fed back to the program to show how the program is developing, including preliminary data on elements related to effectiveness. Column E focuses on summative evaluation based on outcomes that measure the extent to which the program was effective in achieving its intended impacts. Within Fig. 2: Column A (pre-implementation history and context) overlies each other logic model category, and invokes domains based on relevant concepts from the Consolidated Framework for Implementation Research (CFIR) [33, 34] and the Practical, Robust Implementation and Sustainability Model (PRISM) [35] as applied to CRH. For example, the evaluation team identified site readiness and other factors affecting site level equivalence as critical to account for in analyses. Context will inform virtually all qualitative and quantitative analysis. The model implies that, for example, site rurality and location in a medically underserved area will be critical to account for across evaluation domains.

- Column B (program inputs) focuses on implementation by periodically documenting program structure, resources, services, and changes over time. This data will inform assessment of implementation fidelity in terms of whether program resources and features were implemented as planned both initially and over time. Resources provided to hubs from CRH program sources and from hubs to spoke sites are identified as labor and capital inputs.
- Column C (program outputs) includes reach and adoption. Reach assesses the extent to which CRH hubs fully and equitably deliver the care they are expected to provide to spoke site patients needing coverage. Adoption focuses on the extent to which the program is accepted by and valued by stakeholders, on the barriers and facilitators to adoption, and on integration of CRH hub staff into spoke site workflow.
- Column D (program outputs) calls for measuring CRH care delivery, coverage of staffing vacancies of different kinds, and duration of coverage. It also calls for qualitative assessment of CRH challenges and successes.
- Column E (program outcomes) calls for summative assessment of CRH effectiveness relative to the program’s pre-identified goals using formal comparative designs. It also calls for assessment of factors related to maintenance or sustainment of the CRH program following its early implementation phase.

### CRH Evaluation aims and questions

Table 1 links the logic model to the evaluation’s three aims (Column A). The full wording of each aim and the specific evaluation questions within each are shown in Appendix 2. Column B links each aim to the timeline for the main evaluation activities related to it; for reporting back to stakeholders on these; and for producing relevant products.

**Table 1:** Clinical Resource Hub (CRH) Evaluation specific aims

A. CRH Evaluation Aim	B. Frequency & Timeline for Main Activities, Reporting, & Expected Products
**Specific Aim #1:** Formative Evaluation of CRH Implementation, Reach, and Adoption	**Main Activities** (2020 – 2024)**:** • Descriptive qualitative and quantitative data collection and analysis **Reporting Frequency:** Yearly **Products (**2021–2024): • Annual report to program leadership • Presentations to CRH Advisory Board, CRH Scientific Advisors, regional stakeholders, and scientific meetings • Manuscripts
**Specific Aim #2:** Formative Evaluation of CRH Effectiveness	**Main Activities** (2022 – 2024)**:** • Descriptive qualitative and pre-post quantitative data collection and formative analyses **Reporting Frequency:** Yearly **Products** (2022 – 2024): • Annual report to program leadership • Presentations to CRH Advisory Board, CRH Scientific Advisors, regional stakeholders, and scientific meetings • Manuscripts
**Specific Aim #3:** Summative Evaluation of CRH Program Outcomes	**Main Activities** (2024 -2026): • Qualitative and quantitative data collection and comparative analysis (spans pre CRH through 2024) • Expert stakeholder panel consensus process on CRH outcomes (2025) **Reporting Frequency:** Final report **Products** (2026): • Final evaluation report • Final presentations and manuscripts

Aim 1 will initiate documentation of implementation, including data on readiness and context in relationship to implementation progress. Analysis of implementation variations and their determinants will be important for understanding implementation and will also contribute to spoke site level matching required for Aim 3. Aim 2 will focus on collecting longitudinal, non-comparative data on formative program effects on patient care, workplace outcomes, and program challenges and successes. These findings will support the comparative data analyses and the data syntheses required for Aim 3. Aim 3 will support summative conclusions regarding CRH effectiveness based on outcomes relevant to major program goals, including achievement of value to stakeholders [36, 37]. Aim 3 will include mixed methods data synthesis as well as quantitative assessment of impacts on patient and workplace outcomes.

### CRH Evaluation study design overview

The evaluation aims to use rigorous but pragmatic approaches including mixed methods. Both qualitative and quantitative designs (see Appendix 3) assume that CRH is a complex national program with contextual influences [38–40], and that the program will necessarily evolve during evaluation.

One design issue is the level of analyses. Given that the effects of CRH are intended to affect the overall functioning of the spoke sites participating in CRH, the design focuses mainly on the local site level (either CRH spoke sites or equivalent sites not receiving services from CRH). For example, the main effects of CRH are expected to derive from the reduction of competing demands on the staff remaining in place after staffing losses, and thus may affect all spoke site providers and teams, including both patients cared for by covering CRH providers and those cared for by usual site providers. We will also consider some analyses that aggregate results to the national level across all 18 regional hubs and their CRH participants. We consider this approach to be secondary however because of the program’s focus on site level outcomes and because of the challenges in controlling for contextual factors that could affect results.

CRH is aimed at achieving acceptable levels of access to care, quality of care, and workplace-related outcomes for spoke sites in which these may have been impacted by staffing deficits. CRH thus aims to deliver care that is equivalent to, rather than better than, usual care. Non-inferiority designs will be used for relevant analyses such as care quality, and interpretation of findings will also integrate the CRH non-inferiority goal.

Qualitative data designs focus on data from interviews at multiple levels. Interviewees will include hub and spoke site front line providers, spoke site clinical leaders, and hub clinical and administrative leaders. Data will also be abstracted from ongoing CRH program document review. Key results will be tracked longitudinally (2019 – 2024) based on repeated interview questions and investigation of initially identified themes over time.

### Data collection and data base development

Table 2 shows expected key evaluation measures and data sources and indicates which data source will be the main one (underlined). It also indicates patient access to care and staff burnout and turnover as primary outcomes. The table indicates measures at a conceptual level; specifications for final measures will be tested for validity and reliability and are in development. Most measures will require data from more than one source and in some cases will require mixed methods development and interpretation. Quantitative measures of utilization, costs, and staffing, as well as measures of patient characteristics, will rely on data from national VA administrative data bases. Measures of CRH patient care experiences, including access, will rely on data from VA national surveys [41, 42]. Implementation measures will rely on CRH program data, on qualitative data from interviews, and on yearly surveys of the 18 hub directors (three surveys have been completed).

**Table 2:** Clinical Resource Hub (CRH) Evaluation key measures & planned data sources ^a^

	**Planned Main Data Sources**			
**Key Measures Needed**	**CRH Program Electronic Records or Documents**	**CRH Evaluation Surveys**	**CRH Evaluation Qualitative Interviews**	**VA Electronic Administrative or Survey Databases**
**Core History & Context (including Readiness and Site Level Equivalence) Measures**				
Hub site characteristics including readiness	X	X		**X**
Spoke site characteristics	X	X	X	**X**
CRH staffing and governance		**X**	X	X
Need for CRH support including staffing gaps	X	X	X	**X**
**Core CRH Program Region-Level Input, Output, & Formative Outcome Measures**				
Features as planned & as implemented		**X**	X	X
Staffing by professional discipline	X	**X**	X	
Labor and capital costs	**X**			X
Reach & adoption	X	X	X	**X**
Enrolled patient utilization and costs	X			**X**
**Spoke Site Level Formative & Summative Outcomes**				
Patient access to care ^b^				**X**
Staff burnout and turnover ^b^				**X**
Patient clinical quality of care				**X**
Staffing gaps and gap coverage				**X**
**Additional Patient Level Outcomes**				
Overall patient satisfaction with CRH care				**X**
Adverse CRH patient outcomes		X	X	**X**

^a^ The main data source for each measure is bolded and underlined

^b^ Primary outcome measures

### Development of a common CRH Evaluation data base

Evaluation teams will continuously identify data elements needed across teams for inclusion into a shared electronic CRH data base. The CRH Core Team will develop common variable definitions for these elements through across-team deliberations. The CRH Core Team analysts will also maintain documentation that links the data elements to aims and evaluation questions. Team-specific data sets will also exist outside of the core; these include variables that address evaluation questions for which a team is responsible but are unlikely to be needed across teams.

### Quantitative data analysis overview

Formative quantitative data analysis will mainly use simple pre-post measures or run charts, emphasizing graphic presentation, and often controlling for key contextual elements. For summative quantitative assessment of CRH effectiveness, teams will use appropriate multivariable regression models within comparative quasi-experimental designs. Analyses will control for nesting at the regional and/or site levels as appropriate.

Internal validity threats are intrinsic to quasi-experimental designs and can be reduced but not eliminated. To avoid confounding due to unmeasured factors related to program implementation and outcomes, we will fit a separate model for each outcome measure using robust interrupted time series or difference-in-difference analytic approaches. To reduce bias due to non-randomized implementation of CRH, we will use latent variable approaches, propensity score matching, or related methods and will control for important contextual factors in all analyses. Finally, we will use timeline data on external and internal CRH program and evaluation events to link repeated measures data to these contextual influences.

### Qualitative data analysis approach

In general, we will follow a previously described rapid qualitative data analysis approach in which qualitative analysts create and cross-validate summaries for each interview, followed by a random 50% audit to check summaries against transcripts for accuracy and completeness, and finally by inductive and deductive summarizing of major themes [22]. Analyses will consider the extent to which the responding subjects are representative relative to the evaluation questions they address. We will also identify and track key qualitative data elements, such as barriers and facilitators, over time.

### Summative evaluation mixed methods approach

The concurrent mixed methods approach we will use focuses on providing a foundation for synthesizing qualitative and quantitative findings to support decision-making as part of summative evaluation. Our approach builds on triangulation [33, 34]. Analyses will assess validity in terms of congruence (similar consistent results) and/or complementarity (expansion or clarification of results) across different data sources [43, 44].

### Communication of evaluation results

Formative results will be summarized yearly in a report that is submitted to CRH program leadership, to the CRH Advisory Board, and to the Office of Primary Care. Evaluation Team investigators will also continuously present findings on CRH program teleconferences, including those for CRH hub leaders, and at national VA and non-VA sponsored conferences, and will contribute data to the CRH program’s yearly or bi-annual reports to the US Congress. The evaluation team will meet biweekly with CRH program leaders, and present results to CRH Hub leaders and/or Advisory Board members at least yearly. We will use mixed method analysis results as the basis for a summative modified Delphi stakeholder panel that includes Veteran patients (see Table 1, Specific Aim #3) [45, 46], and identifies CRH sustainability measures and strategies. [47] Publication of evaluation results, both formative and summative, will rely substantially on the Standards for Quality Improvement Reporting Excellence (SQUIRE) [48, 49] and the Template for Intervention Description and Replication (TIDieR) [50] guidelines; the protocol process aims to ensure that these guidelines can be met. Use of other implementation science frameworks, including RE-AIM [51], CFIR [34] and PRISM [52], will also be encouraged. All publications will be reviewed for accuracy and adherence to PCAT guidelines and procedures by the CRH core team.

### Anticipated findings

We expect that the evaluation will result in formative findings related to each element of the logic model, and that these will lead to CRH program improvements. We expect that quantitative summative evaluation findings will show whether receiving CRH support enables local sites to improve Veteran access to VA primary and mental health care, and whether CRH achieves this by providing care that is equivalent in quality and cost to care provided by similar comparison sites. We also expect qualitative summative evaluation findings to provide a clear picture of program implementation, including program features, variations, successes, and challenges.

## Discussion

This paper describes the protocol for evaluating a nationally implemented, regionally based VA contingency staffing program for improving patient access to care. The program aims to provide time-limited primary care and mental health staffing, primarily through telehealth modalities, in support of over 1000 VA outpatient sites. The planned evaluation addresses the dearth of studies on contingency healthcare staffing support by describing and assessing the program’s implementation and outcomes.

Health services researchers are increasingly asked to evaluate newly implemented healthcare delivery system programs or policy changes [13, 53]. Large scale implementation is often driven by organizational or political priorities, such as those identified by the US Congress in the case of CRH, and by associated tight timelines that prevent full program or evaluation design prior to implementation. Rigorous program evaluation has the potential to support improved implementation within learning healthcare systems [6, 7, 13, 14] by providing both data showing needed program course corrections during implementation and data aimed at supporting evidence-based decision-making about whether or how program should be maintained once implementation is complete. Yet while extensive guidance on developing protocols for tightly controlled studies such as randomized trials abound, practical guidance on exactly how to develop a protocol for a new and evolving health system-driven program is uncommon [54, 55]. Our protocol development process aimed to use implementation science frameworks to integrate program stakeholder perspectives into a scientifically valid and feasible learning health system-oriented evaluation plan.

We expect that publication of our protocol will maximize the validity and utility of our conclusions as documented in subsequent CRH evaluation publications. Both formative and summative evaluation components emphasize goals, concerns and primary and secondary outcomes identified by CRH program leaders and stakeholders during the initial CRH implementation period. The protocol can thus help the evaluation to maintain a strong focus on what program developers and implementers specified as their intended accomplishments.

Our protocol addresses contingency staffing issues that, based on literature review and early input from CRH implementers, are likely to affect any contingency staffing initiative. We therefore expect that our systematic protocol development process will have supported the relevance and comprehensiveness with which we can inform VA and other learning healthcare systems about contingency staffing in healthcare. For example, our literature review showed that provision of virtual care from staff hired from outside of a local site may be attractive from a variety of perspectives. However, potential negative impacts on patient care and workplace outcomes can occur [56]. Negative impacts could occur in CRH due either to lack of integration of hub contingency staff into spoke site supervision and workflow, or to the substitution of telehealth visits for needed in-person care [57–59]. The protocol calls for data to address these issues, including documenting the type and duration of staffing support provided.

We recognize limitations inherent in our plan. First, our non-randomized study design will not yield definitive information about a causal link between implementation of the CRH intervention and the summative outcomes we identify. Second, in an evolving and growing program, there will ongoing evolution of key data elements. To address this, we will define alternative variable definitions and conduct sensitivity analyses when indicated. We will also account for pandemic-related changes in overall VA use of virtual care by accessing national VA data bases from 2017 onward for CRH supported and non-CRH supported sites. Third, although we will account for key characteristics of sampled sites, staff and Veterans, our ability to assure site-level equivalence for quantitative analysis or for developing representative samples for qualitative work will be limited. Fourth, the evaluation engages Veteran perspectives through qualitative interviews and surveys, rather than direct participation, until 2025, when we will engage them in a panel for considering summative evaluation data (see Table 1). Fifth, early CRH evaluation data suggests that there may be differences between mental health and primary care telehealth modality use [60]. We will address these and other important differences across the two types of services through further protocol development. Sixth, our evaluation focuses on the primary care and mental health components of CRH. These components were the first to be implemented. Engagement of other specialties as part of the CRH program is planned for the future and will be the subject of a separate program planning and evaluation effort. Finally, additional challenges are sure to arise as we implement the evaluation plan. We will account for these in study records and in relevant publications. Assessment of implementation strategies used and of efforts related to CRH sustainment are two areas in which we expect to further develop data collection and analysis as the CRH program matures.

We also recognize potential strengths of our evaluation. By showing how researchers and health systems leaders partnered to integrate the aims, capabilities, and perspectives of each into a final protocol, this paper supports continued development of approaches for achieving learning health systems goals in VA and other large healthcare systems. The protocol development process used a systematically applied researcher and clinical operations partnership model to ensure coverage of both scientific and stakeholder goals. Use of implementation science frameworks, data triangulation from multiple sources, and systematic transition from a formative to summative focus may enable program improvement while driving toward final health system decisions. Finally, engagement of evaluation teams with different focuses during protocol development enables national scale data collection and analysis while the engagement of a coordinating core team maximizes evaluation coherence and synergy.

In summary, we developed an evaluation protocol, informed by implementation and contingency staffing theory, in partnership with delivery system and program leaders who are committed to the concept of VA as a learning organization. Publication of our protocol is aimed both at promoting the integrity, validity, and usefulness of our findings and at sharing our lived example with others interested in partnership evaluation in real-world settings.

## Supplementary Information

**Additional file 1: Appendix 1.** Clinical Resource Hub Program 2019 Congressionally Approved Roadmap.

**Additional file 2: Appendix 2. **Clinical Resource Hub Program Evaluation Specific Aims and Evaluation Questions (EQs).

**Additional file 3: Appendix 3.** CRH Evaluation Design Based on Logic Model Inputs, Outputs, and Formative and Summative Outcomes.

## Data Availability

Not Applicable.

## Abbreviations

VA: Veterans Affairs
CRH: Clinical Resource Hub
OPC: VA Office of Primary Care
PCAT: Primary Care Analytics Team
VISN: Veterans Integrated Service Network
CBOC: Community Based Outpatient Clinics
RE-AIM: Reach, Effectiveness, Adoption, Implementation, and Maintenance
MISSION Act: Maintaining Internal Systems and Strengthening Integrated Outside Networks Act
KN: Kari Nelson
CW: Chelle Wheat
IC: Idamay Curtis
BF: Brad Felker
PK: Peter Kaboli
AR: Ashok Reddy
LR: Lisa Rubenstein
SS: Susan Stockdale
JY: Jean Yoon
LR: Lisa Rubenstein
DG: David Grembowski
R&D: Research and Development
CFIR: Consolidated Framework for Implementation Research
PRISM: Practical, Robust Implementation and Sustainability Model
SQUIRE: Standards for Quality Improvement Reporting Excellence
TIDieR: Template for Intervention Description and Replication
UCLA: University of California Los Angeles
HSR&D: Health Services Research and Development
CADRE: Center for Access and Delivery Research and Evaluation
EQ: Evaluation Question
