# Supplementary material for: Learning from national implementation of the Veterans Affairs Clinical Resource Hub (CRH) program for improving access to care: protocol for a six year evaluation
Source: BMC Health Serv Res. 2023 Jul 25;23:790. doi: 10.1186/s12913-023-09799-5 (PMC10367243; doi:10.1186/s12913-023-09799-5)
Supplement: Supplementary file 1 — Additional file 1: Appendix 1. Clinical Resource Hub Program 2019 Congressionally Approved Roadmap. [file 12913_2023_9799_MOESM1_ESM.docx]

**Appendix 1.** Clinical Resource Hub Program 2019 Congressionally Approved Roadmap

| **Requirement** | **Time Frame of When Requirement is to be Achieved** |
| --- | --- |
| The Nationally Designated Clinical Resource Hub (ND-CRH) is a Veterans Integrated Service Network (VISN) owned resource, with VISN-level governance, providing core services to any facility within the VISN, either virtually or in-person* depending on the clinical service delivery model. | - Existing operational hubs achieve a VISN level governance structure by October 1, 2021. - New hubs have established the governance structure and seen their first patient through the CRH by October 1, 2021. |
| ND-CRH submits metrics on a predefined schedule and upon request. | - Process to achieve this requirement is met and functioning immediately. |
| All facilities within a network utilize a single, standardized online request tool for ND-CRH services. | - Process to achieve this requirement is met and functioning by October 1, 2021. |
| The ND-CRH provides, at a minimum, clinical primary care and mental health services.  Primary care services will include team-based care with primary care providers, PACT clinical pharmacy specialists, primary care mental health integration staff and registered nurses.  Mental health services will include general mental health services. | - Process to achieve this requirement is met and functioning within 2-3 years. |
| Clinical services provided by the ND-CRH are directed to the areas of greatest need. | - Process to identify sites of greatest need in a VISN is required to be in place by October 1, 2021. - Process to achieve additional in-person deployment capability requirement is met and functioning within 2-3 years. |
| Staff within a ND-CRH provide Telehealth Emergency Management (TEM) support when activated and appropriate. | - Process to achieve this requirement is met and functioning within 1 year. |
| ND-CRH supports provider requirements for clinical contact centers. | - Process to achieve this requirement is met and functioning within 3 years. |
| ND-CRH provides services to Veterans at non-VA sites of care, including in the home, on mobile devices and at Advancing Telehealth through Local Access Stations (ATLAS) locations (non-VA sites of care). | - Process to achieve this requirement is met and functioning within 3 years. |
| ND-CRH supports implementation of the new electronic medical record system at transitioning facilities. | - Process to achieve this requirement is met and functioning within 3 years. |
| ND-CRH offers opportunities for graduate medical and associated health education in an effort to foster recruitment and retainment of highly trained providers and increase telehealth expertise. | - Process to achieve this requirement is met and functioning within 3 years. |

**Existing V-IMPACT/Teleprimary Care model includes a portion of in-person care*
