# Supplementary material for: Learning from national implementation of the Veterans Affairs Clinical Resource Hub (CRH) program for improving access to care: protocol for a six year evaluation
Source: BMC Health Serv Res. 2023 Jul 25;23:790. doi: 10.1186/s12913-023-09799-5 (PMC10367243; doi:10.1186/s12913-023-09799-5)
Supplement: Supplementary file 2 — Additional file 2: Appendix 2. Clinical Resource Hub Program Evaluation Specific Aims and Evaluation Questions (EQs). [file 12913_2023_9799_MOESM2_ESM.docx]

**Appendix 2.** Clinical Resource Hub Program Evaluation Specific Aims and Evaluation Questions (EQs)

**Specific Aim #1:** Formative Evaluation of **CRH inputs** (historical and contextual program basis) and **CRH outputs** (program structures and services) as planned and as implemented, summarized yearly through year 4 (2023). (**Context, Implementation Fidelity**)

- **EQ 1.1:** What differences in the internal and external contexts of the 18 CRH regional centers (hubs) and the linked primary care sites (spokes) were there at baseline (2018-2019) that may have influenced ***readiness*** to adopt CRH? Over time? **(CONTEXT)**
- **EQ1.2:** What planned features of ***CRH structure*** (governance, staffing, staff roles, locations, communication) and CRH patient care services (PC and MH service delivery) are achieved during each year between 2020 and 2023? **(IMPLEMENTATION FIDELITY)**
- **EQ 1.3:** What patient and spoke site characteristics predict ***variations in implementation*** fidelity by 2023? **(IMPLEMENTATION FIDELITY)**

**Specific Aim #2:** Formative CRH program achievements and effects, summarized for stakeholder feedback yearly (2020 – 2023) (Implementation Impacts, Reach, & Adoption).

- **2.1 EQ:** How successful is the CRH program in reaching its ***target of dissemination*** across all VISNs and high need spoke sites nationally? **(REACH)**
- **2.2 EQ:** Did CRH clinical staff carry an ***appropriate workload*** in terms of the number of patient encounters completed per assigned workday a CRH spoke site? **(IMPACTS)**
- **2.3 EQ:** Did CRH contingency staff ***integrate into the workflow*** of existing spoke site primary care or mental health teams and standard operating procedures? Did stakeholders at relevant organizational levels have favorable or unfavorable opinions about the CRH Initiative? **(ADOPTION)**
- **2.4 EQ:** How did CRH-associated ***utilization and patient costs*** of care change over time? Do spoke sites that receive CRH support show improved coverage of staffing gaps? Do patients receiving CRH support receive acceptable quality of care? **(FORMATIVE OUTCOMES)**

**Specific Aim #3:** **Summative Evaluation of CRH Outcomes.** The summative evaluation is comparative for effectiveness outcomes, descriptive for maintenance outcomes. Further summation will rest with an expert stakeholder panel that will review results.

- **3.1 EQ:** Did ***clinical staffing at spoke sites*** improve compared to equivalent (e.g., in terms of context, need) sites? Were gaps reduced, and was provider and/or staff loss reduced [primary outcomes] **(SUMMATIVE EFFECTIVENESS)**
- **3.2 EQ:** Is ***patient access*** to care improved in spoke sites? [primary outcome] **(SUMMATIVE EFFECTIVENESS)**
- **3.3 EQ:** Do patients receiving CRH support show equivalent ***clinical quality of care*** compared to same-site, similar patients not in CRH? [secondary outcome] **(SUMMATIVE EFFECTIVENESS)**
- **3.4 EQ:** Are ***utilization and costs*** of care equivalent between comparable same site patients receiving and not receiving hub support? **(SUMMATIVE EFFECTIVENESS)**
- **3.5 EQ:** Were ***disparities*** (racial/ethnic, rural/urban, gender) in quality of care similar or different at CRH spoke sites versus other comparable sites? Put another way, did CRH close or widen or have no impact on racial/ethnic disparities in quality of care? [secondary outcome] **(SUMMATIVE EFFECTIVENESS)**
- **3.6 EQ:** Were there unanticipated negative consequences of CRH?
- **3.7 EQ:** What are the expected costs of maintaining CRH? (**Maintenance**)
- **3.7 EQ**: CRH program leader, hub leader, and participating spoke site ***perceptions of CRH*** based on multi-method evaluation results: were there actual or potential negative and/or positive effects of CRH? Is CRH now considered part of routine care? (**Maintenance**)
- **3.8 EQ:** Can *stakeholder panels* held in evaluation year 3 and year 6 come to consensus on the value of the CRH program? (**Maintenance)**
