# Supplementary material for: Learning from national implementation of the Veterans Affairs Clinical Resource Hub (CRH) program for improving access to care: protocol for a six year evaluation
Source: BMC Health Serv Res. 2023 Jul 25;23:790. doi: 10.1186/s12913-023-09799-5 (PMC10367243; doi:10.1186/s12913-023-09799-5)
Supplement: Supplementary file 3 — Additional file 3: Appendix 3. CRH Evaluation Design Based on Logic Model Inputs, Outputs, and Formative and Summative Outcomes. [file 12913_2023_9799_MOESM3_ESM.docx]

Appendix 3: CRH Evaluation Design Based on Logic Model Inputs, Outputs, and Formative and Summative Outcomes

| **Logic Model Elements** | **Qualitative** | | **Quantitative** | |
| --- | --- | --- | --- | --- |
|  | **Design** | **Data Collection & Analysis** | **Design** | **Data Collection & Analysis** |
| **Context and Inputs:** | | | | |
| **Baseline readiness & pre-program history** | Descriptive | Regional hub analysis based on baseline interviews and program records | Descriptive, multi-method | - Integrates relevant qualitative findings as variables into the CRH evaluation core data base |
| **Program governance, planned and unplanned services and features, staffing patterns** | Descriptive | Longitudinal, regional hub analyses based on periodic (yearly) all-hub interviews and observational analysis of implementation barriers and facilitators based on analysis of themes | Observational, multi-method | - Longitudinal, regional hub analyses based on periodic all-hub surveys and on periodic assessment of CRH program staffing records and qualitatively derived scores or variables |
| **Program labor and capital investments** | NA | NA | Observational | - Longitudinal, regional hub data analysis based on periodic assessment of hub labor costs, funding |
| **Implementation variations** | NA | NA | Observational | - Regression or cross-case analysis of regional variations in hub implementation achievement (dependent variable) based on readiness and context features (independent variables) after completion of initial implementation efforts (Evaluation Year 3) |
| **Outputs:** | | | | |
| **Program reach** | NA | NA | Observational | - Longitudinal assessment of achievement of all key program features across hubs - Longitudinal assessment of provision of hub services relative to regional site gaps in staffing - Analysis of the distribution of spoke sites in terms of the proportion of their assigned patients who receive CRH care over the course of a year (penetration) - Analysis of the relationship between penetration and staffing gaps documented through analysis of data on site-level staffing over time |
| **Program implementation impacts: quantity and types of care delivered** | NA | NA | Repeated measures | - Longitudinal assessment of national utilization (by care modality) of CRH services over time |
| **Program Adoption: Acceptability of key CRH features to stakeholders; stakeholder perceptions of program quality and costs; integration of hub staff into spoke site teams** | Observational | Analysis of acceptability based on - key stakeholder interviews (Evaluation Years 3 and 5) including hub and representative spoke leaders and staff | NA | NA |
| **Program adoption: barriers and facilitators** | Observational | Longitudinal, regional hub analyses based on identification of themes in periodic (yearly) all-hub interviews and | NA | NA |
| **Formative Program Outcomes:** | | | | |
| **Patient utilization and costs for CRH services** | NA | NA | Repeated measures | - Quantity, types of clinical services (modalities, encounter types) over time |
| **Hub workload efficiency** | NA | NA | Repeated measures | - Longitudinal assessment of hub workload (utilization) versus staffing (labor) costs |
| **CRH patient access to and quality of care** | NA | NA | Repeated measures | - Longitudinal assessment of national VA data on patient access experiences (based on monthly surveys) and quality of care measures for patients cared for by CRH |
| **CRH staffing service delivery** | NA | NA | Observational | - Degree to which spoke site staffing gaps are filled by CRH in a timely manner - Duration of gap fulfillment per spoke site - Use of locum tenens (VA or non-VA staff) |
| **CRH Challenges and Successes** | Observational | Qualitative synthesis of themes showing challenges or successes (in Evaluation Year 3) | NA | NA |
| **Summative Program Outcomes:** | | | | |
| **Effects of CRH Participation on Patients** | NA | NA | Quasi-experimental | - Non-inferiority comparison of access and clinical quality of care for CRH spoke site patients versus equivalent sites not receiving CRH support |
| **Workplace outcomes and CRH Maintenance** | Observational | Analysis of qualitative data on workplace effects of CRH, including attitudes toward program maintenance, program stability and equitability, and overall desirability of access to the CRH program | Quasi-experimental, multi-method | - Comparison of staff burnout, morale and turnover between CRH spoke site staff versus equivalent sites not receiving CRH support |
| **Return on Investment** | Qualitative Synthesis; Modified Delphi Stakeholder Panel Synthesis | Integration of qualitative and quantitative findings pertaining to the value of maintaining the CRH program, considering its successes, costs, and benefits | Multi-Method Synthesis of Quasi-Experimental and Qualitative Results | - Integration of findings on program outcomes to develop a synthetic measure of program benefits, and analysis of these in relationship to program labor and capital costs |
